# Nucleic acid detection in the diagnosis and prevention of schistosomiasis

**DOI:** 10.1186/s40249-016-0116-y

**Published:** 2016-03-30

**Authors:** Ping He, Lan-gui Song, Hui Xie, Jin-yi Liang, Dong-ya Yuan, Zhong-dao Wu, Zhi-yue Lv

**Affiliations:** Department of Parasitology, Zhongshan School of Medicine, Sun Yat-sen University, Guangzhou, 510080 China; Key Laboratory of Tropical Disease Control, Ministry of Education, Guangzhou, 510080 China; Department of Pathogenic Biology, Medical School, Xizang Minzu University, Xianyang, 712082 China

**Keywords:** *Schistosoma japonicum*, *S. mansoni*, *S. haematobium*, Diagnosis, Nucleic acid detection

## Abstract

Schistosomiasis is an important zoonotic parasitic disease that causes serious harms to humans and animals. Surveillance and diagnosis play key roles in schistosomiasis control, however, current techniques for surveillance and diagnosis of the disease have limitations. As genome data for parasites are increasing, novel techniques for detection incorporating nucleotide sequences are receiving widespread attention. These sensitive, specific, and rapid detection methods are particularly important in the diagnosis of low-grade and early infections, and may prove to have clinical significance. This paper reviews the progress of nucleic acid detection in the diagnosis and prevention of schistosomiasis, including such aspects as the selection of target genes, and development and application of nucleic acid detection methods.

**Electronic supplementary material:**

The online version of this article (doi:10.1186/s40249-016-0116-y) contains supplementary material, which is available to authorized users.

## Multilingual abstracts

Please see Additional file 1 for translations of the abstract into the six official working languages of the United Nations.

## Introduction

Schistosomiasis or bilharzia, a serious parasitic disease (third in importance to malaria and amoebiasis) caused by blood flukes, continues to plague many developing countries in the tropics. According to the World Health Organization (WHO) [1], in 2012 there were still 240 million patients with schistosomiasis globally, of which 45.8 % were children aged between 5 and 14 years, with 93 % of patients living in Africa. These statistics show little change from those reported previously [2, 3], indicating that schistosomiasis has not yet been effectively controlled.

Human schistosomiasis is mainly caused by three species of schistosomes: *Schistosoma japonicum, S. mansoni*, and *S. haematobium*. A schistosome has a complex life cycle. The adult worms parasitize in the host’s blood system, and their eggs are excreted through human feces or urine. Then, the eggs hatch into miracidia in freshwater sources and quickly penetrate into intermediate host snails, which can subsequently become sporocysts and develop into cercaria by asexual reproduction. Humans acquire the infection after direct contact with cercaria released from infected snails. Different species of schistosomes share similar epidemic patterns.

Accurate surveillance and diagnosis play key roles in the prevention and control of schistosomiasis. Firstly, information on snail infection and cercariae distribution is required in order to evaluate the risk of infection. Both cercarial shedding and microscopic examination of snails are conventional diagnosis methods. However, the positive rate of snails achieved using etiological methods significantly decreases in endemic areas [4]. Moreover, an etiological method is unable to distinguish between the different species of cercaria. The Kato-Katz technique is still the gold standard in schistosomiasis diagnosis. This technique is cheap, convenient, and qualitative [5], but is limited in the diagnosis of low-grade and prepatent infections, as well as in evaluating drug therapeutic effects. The vast increase in genome data for parasites offer valuable insights into the development of novel drug candidates and more accurate diagnostic tools [6]. Some researchers have reported that certain nucleic acid sequences derived from schistosomes can be detected in human sera [7, 8], suggesting that nucleic acid detection may have equivalent diagnostic value as direct parasite detection. Accordingly, the detection of nucleic acid is becoming one of the research foci in the diagnosis of schistosomiasis. Different nucleic acid detection methods have been developed, permitting accurate detection using various samples: infective snails tissue [9–14], cercaria in contaminated water [15, 16], and excretion and blood sample from humans [17–23] and animals [7, 24–27].

Furthermore, nucleic acid-based detection has been shown to be superior in identifying different schistosomes in cases of low-grade infections. The rate of positivity is dramatically higher than that when using the Kato-Katz method and miracidium hatching [8, 12, 18, 28–30]. Deoxyribonucleic acid (DNA)-based assays remarkably reduce the false-negative rate and effectively monitor potential exposure to schistosomiasis. Although immunological methods have comparable sensitivity and specificity as the polymerase chain reaction (PCR) technique, immunological methods cannot distinguish between current infections and past infections [24], which may lead to inappropriate treatment intervention. In contrast, PCR can not only precisely evaluate drug therapeutic effects [7, 27, 31–33], but can also identify early infections [7, 9–11, 25, 34–36] and assess infection density in hosts [37].

This paper reviews the various methods of nucleic acid detection and their sensitivities and specificities in the diagnosis of schistosomiasis.

## Review

### Target sequences of nucleic acid derived from schistosomes

‘Target sequence’ refers to a candidate DNA sequence in a polymerase chain reaction. An applicable target sequence is required for the development of PCR methods. Firstly, the sequence must be species-specific and highly conserved to guarantee the method’s specificity and avoid false-positive results. Secondly, the sequence should contain considerable copies. A greater abundance of the target gene improves the detection limit, enabling detection of trace amounts in a sample and decreasing the false-negative rate. Currently, there are several different target genes used in nucleic acid detection. These are summarized in Table 1.

**Table 1:** Target sequences used for detecting schistosomiasis in humans

*Target sequences*	*Method* ^*[Ref.]*^	*Detection limit*	*Sensitivity %*	*Specificity %*
Repetitive sequences				
SjCHGCS19	Nested PCR [41]	2.02 copies	97.67	96.07
SjR2 (AF412221)	PCR [24]	0.8 pg	60	100
	Real-time PCR [38]	44.7 copies	—	—
	LAMP [7]	0.08 fg	96.7	100
	Nested PCR [40]	—	—	—
SM1-7 (M61098)	PCR [22, 67]	1 fg	96.7	88
	Real-time PCR [26]	0.02 egg	—	—
	LAMP [10]	0.1 fg	—	—
	Touchdown PCR [43]	10 fg	—	—
Dra I (DQ157698)	PCR [44]	10 fg	—	—
	Real-time PCR [23]	0.5 EPG	—	—
	LAMP [10]	0.1 fg	—	—
Ribosomal DNA				
18 s rDNA	Nested PCR [46]	10 fg	—	—
	PCR [47]	40 pg/ul	—	—
	Real-time PCR [25]	0.01 pg	—	—
28 s rDNA	Multiplex PCR [48]	—	94.4	99.9
	PCR [49]	—	—	—
	LAMP [9]	100 fg	—	—
SSU-rDNA (X53047)	Real-time PCR [50]	10 fg	—	—
Mitochondrial DNA				
NADH I	Real-time PCR [51]	1 EPG	—	—
NADH-3	PCR [55]	1 pg	—	—
COX I	PCR [56]	0.01 pg	—	—

*EPG* eggs per gram of feces

“—”: no mention or no accurate data in paper

#### Repetitive sequences

Repetitive sequences are highly repetitive DNA sequences located in a chromosom. An example is SjR2 [GenBank accession no: AF412221], a non-long terminal repeat retrotransposon first isolated from *S. japonicum* genome by Laha [38]. Hybridization analyses have indicated that SjR2 were dispersed throughout *S. japonicum* chromosomes. SjR2 is about 3.9 kb in length with a high copy number (about 10,000 copies per cell), accounting for up to 14 % of the nuclear genome. Various Chinese researchers have characterized this sequence using various methods: PCR [24], real-time fluorescent quantitative PCR [37], loop-mediated isothermal amplification (LAMP) [7], and nested PCR assay [39]. Results showed that no specific amplification occurred in *S. mansoni* [24], *Paragonimus westermani*, *Clonorchis sinensis*, or *Trichinella spiralis* [37]. Thus, SjR2 is an ideal target sequence for *S. japonicum* nucleic acid detection. Similarly, a new 303 bp sequence from another repeat retrotransposon (SjCHGCS19) also showed high sensitivity and specificity to *S. japonicum*. A nested PCR technique based on SjCHGCS19 was developed and successfully applied to detect serum samples from rabbits and human patients [40].

SM1-7 [GenBank accession no: M61098] is a highly repeated, tandemly arranged DNA sequence from *S. mansoni*, which contains 121 bp tandem repeats (600,000 copies per cell) and comprises at least 12 % of *S. mansoni* genome of both sexes [41]. Based on this sequence, PCR [22], real-time PCR [26], LAMP [10], and touchdown PCR [42] methods have been established. Sensitivity tests have shown that there is no cross-reaction with *S. haematobium, S. magrebowiei* [41], *Ascaris lumbricoides*, *Ancylostoma duodenale*, *Taenia solium*, *Trichuris trichiura* [22], *S. intercalatum*, *Trichobilharzia ocellata*, *Apatemon minor*, *Diplostomum spathaceum*, *Echinostoma caproni*, *E. revolutum*, *Hypoderaeum conoideum*, and *Isthmiophora melis*. However, a cross-reaction was observed with *S. rodhaini* [15]. Hence, the SM1-7 sequence is an ideal target gene of *S. mansoni*.

Dra I [GenBank accession no: DQ157698] is a tandem repeat sequence of *S. haematobium* genomes. It is also 121 bp in length and contains a *Sau3A* restriction site, accounting for 15 % of the entire genome. It was first obtained by Hamburger using a PCR method [43]. Studies confirmed that this approach enabled the detection of single cercaria, indicating that it’s a potential tool for the detection of schistosome-infected water sources. A specificity study found that Dra I does not hybridize with *S. mansoni*, *T. ocellata*, and *Echinostoma spp.*, but can hybridize with *S. mattheei*, *S. magrebowiei*, *S. bovis*, *S. curassoni*, and *S. intercalatum*. To overcome the poor specificity of Dra I, a combined detection methodology, based on Dra I and the repetitive sequence SH73 [44], has been established, which can accurately discriminate between the trematodes mentioned above. Additionally, PCR [43], real-time PCR [23], and LAMP [10] targeted at Dra I have been developed and applied to detect excrement and serum DNA from humans and snails, indicating its potential application in the diagnosis of schistosomiasis.

#### Ribosomal DNA

The ribosomal DNA (rDNA) sequence is highly repetitive and conserved, and enables the encoding of ribosomal RNA (rRNA). The 18S and 28S subunits have been applied in the detection of nucleic acid sequences of schistosomes. As early as in 1997, a nested PCR technique based on 18 s rDNA was developed and successfully used to detect a single sporocyst just 1 day after infection with *S. mansoni* [45]. The detection limit has been shown to be 10 fg. This approach can effectively monitor infected snails. Using the 18 s rDNA of *S. japonicum*, a simple and rapid PCR assay was developed and applied to identify infected *Oncomelania hupensis* snails [46]. Similarly, a real-time PCR technique also based on 18 s rDNA gene of *S. japonicum* was established and used to detect parasite-derived DNA in mouse feces and serum samples [25]. The detection limit was 10 fg, which is 100 times more sensitive than that arrived at using conventional PCR. No cross-reaction was found with *S. mansoni*, *C. sinensis*, and *P. westermani*; the exception was *S. sinensium*. However, this target sequence shows high similarity with *S. malayensis*, *S. mekongi*, and *S. sinensium* using alignment software, which indicates that this method is potential tool both for monitoring infection with Asian schistosomes and for early diagnosis of schistosomiasis. Additionally, a powerful multiplex PCR technique based on 28 s rDNA of several schistosomes has been developed and applied in the detection of schistosomiasis using human urine specimens [47]. The sensitivity and specificity is up to 94.9–100 % and 98.9–99.9 %, respectively. Meanwhile, a PCR assay, also based on 28 s rDNA, has been established and successfully used to detect nucleic acid in mice feces [48]. In another study, a real-time PCR method based on the small subunit (SSU) rDNA [GenBank accession no: X53047] was established and able to detect as little as 10 fg of feces [49]. No cross-reaction was found with *E. paraensis*, *S. haematobium*, *S. japonicum*, *S. rhodaini*, *Cercaria minensis*, *C. macrogranulosa*, and *C. caratinguensis*, indicating satisfactory specificity and sensitivity. Using the 28 s rDNA sequence [GenBank accession no: Z46504] of *S. japonicum*, PCR and LAMP methods were able to detect 362 snails in endemic areas, showing these methods’ potential for monitoring infected water sources [9]. In 2015, a LAMP assay successfully detected *Oncomelania hupensis* snails collected from 28 endemic areas, demonstrating no cross-reaction with *P. westermani*, *C. sinensis*, *S. mansoni*, and *Angiostrongylus cantonensis* [50].

#### Mitochondrial DNA

Mitochondrial DNA is a circular DNA molecule that exists in eukaryotic cells. It is highly conserved and repetitive (thousands of copies per cell), which means it is also an ideal target sequence. A novel real-time PCR technique targeted at nicotinamide adenine dinucleotide hydrogen (NADH) I (mitochondrial NADH dehydrogenase I gene) was developed [51], which was specific for *S. japonicum* when tested against *S. mansoni*, *S. haematobium*, *S. bovis*, *T. trichiura*, *A. duodenale*, and *Taenia spp*.. This method was successfully applied to detect *S. japonicum* DNA in human stool samples [28, 52, 53] with low-intensity infections, and in pig feces [54]. Recently, a specific and efficient PCR assay based on NADH-3 [GenBank accession no: KF834975] was described and successfully used to detect snails and urine samples of patients infected with schistosome [55]. This approach can accurately differentiate *S. haematobium* from *S. bovis*, *S. mattheei*, *S. curassoni*, *S. intercalatum*, and *S. magrebowiei*, indicating a potential application in the identification of a coinfection.

COX I is a mitochondrial gene encoding cytochrome C oxidase subunit I. A powerful PCR assay targeted at COX I of several human schistosomes was developed, which was able to identify and differentiate *S. mansoni*, *S. haematobium*, *S. japonicum*, and *S. mekongi* infections [56].

### Development and application of nucleic acid detection in the diagnosis of schistosomiasis

#### Nucleic acid detection of *S. japonicum*

Among the three main species of human schistosomes, *S. japonicum* causes the most serious damages to the hosts. This species is endemic in Asia, primarily in China, the Philippines, and Indonesia [3]. Its intermediate host is *Oncomelania hupensis*. The latest epidemiological investigation showed that there were approximately 300,000 schistosomiasis patients in China by the end of 2012 [57]. Thus, accurate monitoring of infected snails and freshwater sources can provide timely data of the epidemic for the local human population. As shown in Table 2, a rapid PCR technique based on the SjR2 sequence was developed [14] and used to analyze cercaria distribution and dynamic changes in mountainous areas of the Sichuan Province. No cross-reaction was observed with *S. mansoni* and in negative snails. A sensitive and specific real-time PCR assay targeted at SJCHGC08270 [GenBank accession no: AY812553] was developed, which enabled for the detection of minute amounts of nucleic acid (equivalent to 0.5 cercaria) in contaminated water sources [16]. No specific amplification was found for *S. mansoni*, *S. haematobium*, avian schistosomes, and organisms present in water samples of non-endemic areas, indicating the potential value of SjR2 and SJCHGC08270 detection in the monitoring of water sources in endemic areas. Recently, a highly sensitive LAMP method using the 28 s rDNA of *S. japonicum* was developed [9]. The detection limit was 100 fg. This approach enabled the detection of DNA from a single miracidium and snail infected with one miracidium 1 day after infection. Moreover, this approach could detect specific DNA from a group of 100 normal snails mixed with one infected snail, and could also detect schistosomal DNA in snails found negative by microscopy.

**Table 2:** Nucleic acid detection of schistosomes and their corresponding applications

*Biomarkers*	*Method*	*Sample type*	*Sensitivity %*	*Specificity %*	*References*
*S. japonicum*					
SjR2 (AF412221)	LAMP	human and rabbit serum	96.7	100	[7]
SjR2 (AF412221)	PCR	rabbit serum	60	100	[24]
SjR2 (AF412221)	Real-time PCR	rabbit serum	—	—	[38]
SjR2 (AF412221)	Nested PCR	rabbit serum	—	—	[40]
SjCHGCS19	Nested PCR	human and rabbit serum	97.67	96.07	[41]
28 s rDNA(Z46504)	LAMP	snail tissue	—	—	[14]
SjCHGC08270	Real-time PCR	Water	93	—	[16]
5D gene	Nested PCR	adult worm and egg	—	—	[58]
NADH I	Real-time PCR	human, pig, buffalo feces	—	—	[28, 51, 52]
Mitochondria gene	PCR	adult worm	—	—	[60]
ITS2	Real-time PCR	mouse feces, snail tissue	—	—	[61]
*S. mansoni*					
SM1-7 (M61098)	PCR	human feces, urine, serum and mice serum	96–100	88–91.2	[17, 18, 22, 35, 67, 70, 71]
SM1-7 (M61098)	PCR	water and plankton	—	—	[15, 65]
SM1-7 (M61098)	LAMP	snail tissue	—	—	[10]
SM1-7 (M61098)	PCR-ELISA	human feces	97.4	85.1	[68, 69]
SM1-7 (M61098)	Real-time PCR	human feces and serum	80	92.4	[8]
Genomic DNA	Hybridization	snail tissue	—	—	[63]
18 s rDNA	Nested PCR	snail tissue	—	—	[46]
mitochondria gene	Multiplex PCR	snail tissue	—	—	[64, 66]
Satellite DNA	LAMP	mice feces and serum	—	—	[72]
*S. haematobium*					
Dra I	PCR	adult worm, snail tissue, human brain and urine	98	82	[12, 13, 20, 44, 73]
Dra I	Real-time PCR	human serum, urine, feces	96.6–100	93.6–94.4	[23]
Dra I	LAMP	snail tissue	—	—	[10]
Sh110	PCR	adult worm, snail tissue	—	—	[74]
Sh73/Dra I	Multiplex PCR	adult worm, snail tissue	—	—	[45]
NADH3	PCR	snail tissue	—	—	[55]
ITS2 (DQ677661)	PCR	human urine	—	—	[33, 34]
Differential detection methods					
ITS2 (AF146037/8)	PCR-RFLP	adult worm and cercaria	—	—	[75]
COX I	Multiplex PCR	adult worm	—	—	[76]
005AAT/Dra I	PCR	adult worm	—	—	[77]
Dra I/SM1-7	PCR	human urine, feces	99–100	71–100	[17]
COX I	PCR	adult worm, human blood	—	—	[56, 78]
Mitochondria gene	Copro-PCR	human feces	87.7	100	[79]

“—”: no mention or no accurate data in paper

Adult worms parasitize in the host’s mesenteric-hepatic portal vein system, and eggs deposited in the intestinal wall are discharged in stool. To date, the Kato-Katz smear method is still the gold standard for the diagnosis of *S. japonicum* infection. To overcome the limitations of low sensitivity and cross-reaction in the traditional stool examination method, PCR and nested PCR methods based on the *S. japonicum* 5D gene (coding miracidium antigen) were simultaneously established for the first time in 1998 [58], enabling a single egg or cercaria to be detected. Studies have demonstrated that this specific method has no cross-reaction with intestinal bacteria and human genome. Later, a series of detection methods based on SjR2 repetitive retrotransposon were established, including PCR [24], real-time PCR [37], LAMP [7], and nested PCR [39]. Among these methods, LAMP’s detection limit was 0.08 fg, which means it’s the most sensitive, about 10^4^ times more so than conventional PCR (LAMP is 96 % as compared with PCR, which is 60 %). Furthermore, this procedure detected *S. japonicum* DNA in rabbit sera one week after infection and 12 weeks post-treatment with praziquantel. These results demonstrate the potential application of LAMP assay in the early diagnosis of, and in the evaluation of therapy effectiveness for, *S. japonicum* infection. In another study, a real-time PCR method targeted at NADH I [51] was able to detect *S. japonicum* DNA in a sample of negative stool mixed with a single egg. No cross-reaction was observed with *S. mansoni*, *S. haematobium*, and *S. bovis*. This method successfully detected a low-intensity infection in 1,727 human stool samples [28] and 12 pig feces [54], even immunoglobulin G antibody titers remained high in all infected groups in the latter study. This same method was also used to check previous observations based on microscopy, to determine that the Philippine buffalo was not a major reservoir host for schistosomiasis [59]. An investigation of 81 random samples of buffalo stool by different methods showed that the prevalence rate of *S. japonicum* infection was 3.7 % by Kato-Katz smear, 0 % by miracidia hatching, and 51.5 % by real-time PCR. Therefore, it is necessary to reevaluate the role of Philippine water buffalos in the transmission of *S. japonicum* infection.

Other highly specific detection methods have also been established. A specific and sensitive PCR procedure based on *S. japonicum* mitochondrial genes [GenBank accession no: NC-002544] was developed. Its detection limit was 0.05 ng and it has the ability to distinguish between closely-related trematode species, such as *S. mansoni*, *S. haematobium*, *Fasciola hepatica*, *F. gigantica*, *C. sinensis*, and *Opisthorchis viverrini* [60]. Another specific PCR technique targeted at internal transcribed spacer (ITS)2 sequence was described and applied to detect infected snails and fecal samples of mice. No cross-reaction was observed with *S. mekongi*, *F. gigantica*, *O. viverrini*, *E. malayanum*, and *Paragonimus heterotremus* [61].

#### Nucleic acid detection of *S. mansoni*

*Schistosoma mansoni* is mainly distributed in Africa, as well as in the Middle East and South America [62]. Its intermediate host is *Biomphalaria*. The conventional identification of snails infected with *S. mansoni* involves observing cercarial shedding under light. Alternatively, snails may be crushed between glass slides and inspected for the presence of sporocysts. However, these detection methods have limitations when there’s a low parasite burden, in prepatent infections, and in concurrent infections. Fortunately, a dot hybridization method was developed to detect *S. mansoni* DNA in snails, which enabled the monitoring of early prepatent and apparent infections [63]. In 1997, a nested PCR method targeting the 18S rDNA of *S. mansoni* was described by Hanelt [45]. The detection limit was 10 fg. Infected snails were detected as early as 1 day after penetration of a single miracidium. In another study, a multiplex PCR approach based on the minisatellite repeat sequence from *S. mansoni* mitochondrial DNA was developed [64], which enabled the identification of infected snails during prepatent infections. In 1998, the use of PCR amplification of *S. mansoni* (SM)1-7 was proposed to detect cercariae of *S. mansoni* in water [65]. This approach could detect as little as 10^−6^ ng of *S. mansoni* DNA and a single cercaria, but it was not widely used because of the PCR product’s ladder pattern. In 2006, a single-step technique based on multiplex PCR assay was developed for the identification of *Biomphalaria* species and diagnosis of *S. mansoni* infection simultaneously [66]. Species-specific primers were directed both at ITS2 of snails and the mitochondrial DNA of *S. mansoni*. The methodology has been shown to be efficient, fast, and reproducible for *Biomphalaria* identification and diagnosis of snails infected with *S. mansoni* during the prepatent period. Recently, a LAMP assay based on the highly abundant sequence SM1-7 was established [10]. Detection limit of this assay is 0.1 fg, which is 10 times higher than that of the PCR method described above. It is possible to identify infected snails from the first day after exposure to miracidia. Test results can be directly assessed by the naked eye, indicating its potential for monitoring early infections and infections in a large number of samples.

Similar to *S. japonicum*, *S. mansoni* adults parasitize in mesenteric blood vessels, and eggs are mainly excreted through feces. Accurate detection of egg-derived DNA is helpful for the differential diagnoses of schistosomiasis. In 2002, a PCR assay was established and used to detect serum and stool specimens from patients infected with *S. mansoni*, revealing no cross-reaction with *A. lumbricoides, A. duodenale, T. solium*, and *T. trichiura* [22]. A sensitivity study showed that the detection limit was 1 fg (10 times lower than that when using the Kato-Katz method). This method was successfully applied to investigate fecal samples of 194 individuals living in an endemic area [67]. The prevalence of infection was 30.9 % in triplicate fecal samples examined using the Kato-Katz method, whereas it was 38.1 % in a single fecal sample examined using the PCR technique. Subsequently, a technique incorporating PCR-enzyme-linked immunosorbent assay (ELISA) based on the former PCR assay was developed and used to detect infection in human fecal samples, showing the potential for estimating parasitic load and therapeutic efficacy [68, 69]. Furthermore, this method was also applied to analyze DNA from human urine samples [17, 70], feces [18, 71], and mice serum [34] by others. Recently, another LAMP method based on *S. mansoni* satellite series [GenBank accession no: L27240] was established and successfully used to detect *S. mansoni* DNA in stool samples as early as 1 week post-infection, whereas the Kato-Katz technique cannot detect eggs in feces until 6 weeks post-infection. No cross-reaction was found with *S. japonicum*, *S. haematobium*, *S. intercalatum*, *T. taeniaeformis*, *E. granulosus*, and other nematodes, which suggests high specificity [72]. Furthermore, Carvalho [26] applied a real-time PCR assay targeted at SM1-7 to examine human feces and serum samples. The results showed that the positive rate arrived at by using the PCR technique was higher than that obtained by the Kato-Katz method. In another study, a similar real-time PCR technique was used to test hamster feces and serum samples [8]. The first detection of *S. mansoni* eggs in feces by the Kato-Katz method and by PCR occurred 49 days post-infection. In contrast, the detection of *S. mansoni* DNA in serum using the PCR technique was detected from 14 up to 56 days post-infection, demonstrating that serum is a trustworthy source of DNA in cases of prepatent infections.

#### Nucleic acid detection of *S. haematobium*

*Schistosoma haematobium* is mainly distributed in Africa, the Middle East, and the Eastern Mediterranean [1]. Adult worms live in the host’s pelvic venous plexus and bladder veins, causing urogenital schistosomiasis. The eggs can penetrate the vessel wall and migrate toward the mucosal tissue and lumen of the genitourinary tract. Some eggs are excreted in urine or genital secretions, while a substantial portion becomes trapped in tissues of urogenital organs and induces acute and chronic inflammation. Hematuria and proteinuria can be used in clinical diagnosis. The gold standard of diagnosis is microscopic detection of eggs in urine, but this method is less sensitive in cases of low-grade infections and also it cannot detect single sex or prepatent infections. A highly sensitive PCR assay using the Dra I repetitive sequence was established, enabling the detection of single cercaria [43]. The detection limit was as little as 10 fg of *S. haematobium* DNA. The specificity test showed that the Dra I sequence cross-reacted with *S. bovis*, *S. magrebowiei*, *S. mattheei*, *S. curassoni*, and *S. intercalatum*, but not with *S. mansoni*, *Trichobilharzia ocellata*, and *Echinostoma* spp.. Consequently, this method has been applied to a variety of specimens by various researchers [13, 73]. For instance, a total of 2,146 *Bulinus globosus* snails were examined by Allan [12]. The positive rate detected using the PCR method (40–100 %) was consistently higher than in the evaluation of shedding cercariae (3.96 %). Similarly, 89 urine specimens from school-age children in Niger were analyzed by Ibironke [20]. The PCR method showed higher sensitivity compared to that observed in the microscopy of eggs (57.3 % versus 49.4 %).

To satisfy the demand of screening prepatent infections and low parasite loads, more sensitive methods have been established. A LAMP method based on the Dra I repeated sequence has been developed [10]. The detection limit (0.1 fg) was 100 times more sensitive than that achieved using the PCR method described earlier [43]. It was possible to identify infection from the first day after exposure to miracidia. Later, a real-time PCR assay also based on Dra I was developed [23], which was successfully applied to detect infection in human feces, serum, and urine samples.

In addition, nucleic acid detection has an advantage in evaluating treatment efficacy. A six-month cohort study [32] was conducted to assess treatment efficacy with single-dose praziquantel (40 mg/kg), for which 33 women with *S. haematobium* infection were recruited. After treatment, eggs were not detected in the women’s urine and cervical samples, however, the PCR method was able to detect schistosome DNA in eight of the women (24 %). This study suggests that single-dose praziquantel cannot completely eliminate *S. haematobium* DNA in a substantial number of patients. In another study, 390 urine specimens from 114 school-age children infected with *S. haematobium* were examined using microscopy and real-time PCR assay [33]. Daily fluctuations in microscopy diagnosis were more significant than those observed using the PCR method. Moreover, the PCR method showed higher sensitivity (92 %) when compared to microscopy (31 %), both pre-treatment and post-treatment. This confirms that the real-time PCR method is an accurate and reproducible tool for monitoring treatment effectiveness.

The *Bulinus* species is an intermediate host of *S. haematobium* and other related trematode species. Moreover, the Dra I sequence cross-reacted with certain animal schistosomes, therefore a more effective tool for accurately monitoring infected snails is required. In 2007, a PCR assay targeting a repetitive sequence, *S. haematobium* (Sh)110, was developed and successfully applied to detect infection in wild snails [74]. This method enabled accurate distinction of *S. haematobium* from *S. curassoni*, *S. intercalatum*, *S. bovis*, and *S. mattheei* (but not from *S. margrebowiei*) through different amplification bands. Subsequently, a better multiplex PCR assay using the specific sequence SH73 and Dra I was established and successfully applied to detect infected wild snails. It was able to differentiate *S. haematobium* from *S. curassoni*, *S. intercalatum*, *S. bovis*, *S. mattheei*, and *S. margrebowiei* [44]. This method may enable the accurate evaluation of infected snails where *S. haematobium* is coexistent with other prevalent schistosome species. Recently, a simple and efficient PCR technique based on the mitochondrial gene NADH-3 [GenBank accession no: KF834975] was described [55]. Its detection limit was shown to be 1 pg and no cross-reactions with other related schistosomes were observed.

### Differential detection methods of schistosomes

Snails are intermediate hosts of schistosomes and many other parasites. Both human and animal schistosomes may cause coinfection in certain endemic areas. Generally, an etiological method or a simple PCR method cannot distinguish between the species of *Schistosoma*, whereas combined detection methods can achieve this.

To date, *S. mansoni*, *S. haematobium*, and *S. bovis* are widespread in Africa. *Bulinus* is the intermediate host of the latter two schistosomes and the morphology of their cercaria is quite similar. A novel PCR-restriction fragment length polymorphism (RFLP) method based on ITS2 enabled the differentiation of *S. haematobium* from *S. bovis* through restrictive enzyme-digested products [75]. Similarly, an efficient multiplex PCR technique based on COX I was developed, which can distinguish *S. haematobium* from *S. bovis* [76]. In another study, a PCR assay based on Dra I sequence of *S. haematobium* and satellite sequence 005AAT of *S. mansoni* was described [77]. The detection limit was 10 fg. Specificity studies showed that 005AAT of *S. mansoni* only had a cross-reaction with *S. rodhaini*, while Dra I sequence had cross-reaction with many animal schistosomes, suggesting the potential limitation of Dra I in the diagnosis of schistosomiasis. Recently, a combined PCR procedure targeting Dra I and SM1-7 was established and applied to analyze 86 human samples. The sensitivity and specificity were 99–100 % and 71–100 %, respectively. Meanwhile, results using the Kato-Katz method for *S. mansoni* (76 %) and haematuria for *S. haematobium* (30 %) were shown to be significantly less sensitive [17].

Endemic schistosome species in Asia include *S. japonicum*, *S. mansoni*, and *S. mekongi*. A combined method was developed to distinguish between the four species. The universal primers and species-specific primers were designed according to COX I and the mitochondria gene, respectively. This method has been tested by analyzing serum and urine specimens of canoidea and murine [56], as well as human blood samples [78]. In the latter study, schistosome DNA was detected from all Kato-Katz-positive samples and partial Kato-Katz-negative samples (9/22 serum and 10/41 urine samples). A multiplex PCR assay targeting mitochondria DNA was used to distinguish *S. mansoni* from *S. japonicum* [79]. Another multiplex PCR based on 28 s rDNA sequence [47] was able to simultaneously differentiate *S. bovis* from other human schistosomes (*S. japonicum*, *S. mansoni*, *S. haematobium*, *S. intercalatum*). This detection procedure was successfully used for human urine specimens. The sensitivity and specificity were 94.9–100 % and 98.9–99.9 %, respectively.

## Conclusion

Schistosomiasis remains one of the most prevalent parasitic infections in the world, and has significant economic and public health consequences, particularly in poor communities. The advent of large-scale mass chemotherapy campaigns for schistosomiasis in endemic countries has led to a lower intensity of infections and consequently reduced the effectiveness of conventional diagnostic tests such as serology and the Kato-Katz stool smear. However, more sensitive detection methods are needed in the clinical setting and for epidemiological studies. With the rapid development of molecular biological techniques, nucleic acid tests show potential for application in the diagnosis of infections. These methods are characterized by high sensitivity, high specificity, and rapid response. Investigators from different countries have developed a variety of nucleic acid detection methods based on various target genes. These have been demonstrated to detect parasite-derived DNA in snail tissue, monitor cercaria in contaminated water sources, provide reliable diagnostic results, evaluate drug therapeutic effects, assess host infection density, and identify early or prepatent infections. Of these methods, the highly sensitive and specific LAMP assay has already been used for amplification of other microorganisms and parasites. Unlike the PCR and real-time PCR techniques, LAMP assay does not require amplification cycles by thermocycling or amplicon detection by electrophoresis. Another advantage is that test results can be assessed by the naked eye. This suggests that the LAMP method may be useful in the detection of schistosomiasis in the field.

Generally, there are limited reports on the application of these detection methods in endemic areas. The fact is that nucleic acid tests require a well-equipped laboratory and highly trained technicians, all of which are much more costly than using etiological methods. DNA extraction is a critical step when carrying out nucleic acid tests. There are different commercial kits for extracting DNA from blood, urine, tissues, and fecal samples. Among the various biological samples, feces pose the most complex problems for DNA extraction and require new strategies to be developed. A simple and inexpensive DNA preparation method is required. It is also easy to contaminate nucleic acid amplification under unsuitable laboratory conditions, thus giving rise to false-positive results. As the gold standard of schistosomiasis diagnosis, the Kato-Katz method has many advantages. It is convenient, rapid, cheap, and less demanding of laboratory facilities. Although a large amount of experimental data has shown that both sensitivity and specificity of nucleic acid tests are significantly higher than those of the Kato-Katz method, the nucleic acid method cannot completely replace Kato-Katz. The procedures of the nucleic acid method should be simplified and the cost should be reduced, thus enabling the development of suitable detection techniques for large-scale application in endemic areas of schistosomiasis.

## Additional file

Additional file 1: Multilingual abstracts in the six official working languages of the United Nations. (PDF 238 kb)

## Abbreviations

COX: cyclooxygenase
DNA: deoxyribonucleic acid
ELISA: enzyme-linked immunosorbent assay
ITS: internal transcribed spacer
LAMP: loop-mediated isothermal amplification
NADH: nicotinamide adenine dinucleotide hydrogen
PCR: polymerase chain reaction
RFLP: restriction fragment length polymorphism
RNA: ribonucleic acid
SH: *Schistosoma haematobium*
SjR2: *Schistosoma japonicum* retrotransposon 2
SM: *Schistosoma mansoni*
WHO: World Health Organization
