# Supplementary material for: Nucleic acid detection in the diagnosis and prevention of schistosomiasis
Source: Infect Dis Poverty. 2016 Mar 30;5:25. doi: 10.1186/s40249-016-0116-y (PMC4812660; doi:10.1186/s40249-016-0116-y)
Supplement: Additional file 1: — Multilingual abstracts in the six official working languages of the United Nations. (PDF 238 kb) [file 40249_2016_116_MOESM1_ESM.pdf]

Translation of the abstract into the six official working languages of the United Nations

كشف الحمض النووي في تشخيص البلهارسيا والوقاية منها

بينغ هـ إي، لان غوي سونغ، هوي زي، جين لي ليانغ، دونغ يا يوان، زونغ داو وو، زي يي ل ف

موجز

البلهارسيا مرض طفيلي هام حيواني المصدر يسبب أضرارا خطيرة لكل من الإنسان والحيوان. تلعب المراقبة والتشخيص دورا رئيسيا في مكافحة البلهارسيا، لكن التقنيات المتوفرة حاليا لمراقبة وتشخيص المرض محدودة القدرة. ومع تزايد البيانات الجينومية للطفيليات، فإن التقنيات الحديثة للكشف عن تسلسلات النيوكليوتيدات المشاركة تحظى باهتمام واسع النطاق. هذه الطرق الحساسة والنوعية والسريعة ذات أهمية خاصة في تشخيص حالات العدوى المبكرة المنخفضة الدرجة، وقد تثبت أهميتها سريريا. يستعرض هذا البحث التقدم في تحري الحمض النووي في تشخيص مرض البلهارسيا والوقاية منه، بما في ذلك جوانب مثل اختيار الجينات المستهدفة، وتطوير وتطبيق طرق كشف الحمض النووي.

Translated from English version into Arabic by Lina SM, through

TRANSLATORS
WITHOUT BORDERS

核酸检测在血吸虫病诊断及预防中的应用前景

贺平，宋兰桂，解辉，梁锦仪，袁东亚，吴忠道，吕志跃

摘要

血吸虫病是一种严重危害人类和其他动物宿主健康的重要人畜共患病。监测和诊断在血吸虫病防治中发挥重要作用，然而，目前现场使用的检测诊断技术仍存不足。随着寄生虫基因组大数据的释放，整合核苷酸序列的新检测技术受到广泛关注。这些灵敏、特异和快速的检测方法对轻度感染和早期感染的诊断尤为关键，并具重要的临床指导意义。本文对核酸检测在血吸虫病诊断和防治中的研究进展进行综述，包括靶基因筛选和核酸检测方法的研发与应用等。

Translated from English version into Arabic by Lv Zhiyue, through

Détection d’acides nucléiques dans le diagnostic et la prévention de la schistosomiase

Ping HE, Lan-gui SONG, Hui XIE, Jin-yi LIANG, Dong-ya YUAN, Zhong-dao WU, Zhi-yue LV

RÉSUMÉ

La schistosomiase constitue une importante maladie zoonotique parasitaire à l’origine de graves dommages pour les êtres humains et les animaux. La surveillance et le diagnostic occupent un rôle clé dans le contrôle de la schistosomiase, mais les techniques actuelles de surveillance et de diagnostic de la maladie ont néanmoins des limites. Face à l’augmentation de la quantité de données génétiques pour les parasites, de nouvelles techniques de détection incorporant des séquences de nucléotides attirent toujours plus l’attention. Ces méthodes de détection

sensibles, spécifiques et rapides sont particulièrement importantes pour le diagnostic d'infections précoces et mineures et pourraient s'avérer significatives d'un point de vue clinique. Le présent article passe en revue les avancées de la détection d'acides nucléiques dans le diagnostic et la prévention de la schistosomiase et notamment certains aspects, tels que la sélection de gènes cibles ainsi que le développement et l'application de méthodes de détection d'acides nucléiques.

Translated from English version into French by eric ragu, through

Обнаружение нуклеиновой кислоты в диагностировании и профилактике бильгарциоза

Ping HE, Lan-gui SONG, Hui XIE, Jin-yi LIANG, Dong-ya YUAN, Zhong-dao WU, Zhi-yue LV

АННОТАЦИЯ

Бильгарциоз — это опасный бактериальный зооноз, который наносит огромный вред людям и животным. Надзор и диагностирование играют ключевую роль в борьбе с бильгарциозом, однако современные методы надзора и диагностирования этого заболевания помогают не всегда. По мере получения новых данных о геноме паразитов, всё больше внимания получают новые методы диагностики, включающие нуклеотидную последовательность. Эти чувствительные, специфические, скоростные методы диагностики особенно полезны для выявления хронических и ранних инфекций и могут иметь клиническую значимость. В настоящей работе рассматриваются достижения в применении методов обнаружения нуклеиновой кислоты для диагностирования и профилактики бильгарциоза, включая такие стороны вопроса, как выбор целевых генов и разработка и применение методов обнаружения нуклеиновой кислоты.

Translated from English version into Russian by Elena Alieva, through

La detección de ácido nucleico en el diagnóstico y prevención de la esquistosomiasis

Ping HE, Lan-gui SONG, Hui XIE, Jin-yi LIANG, Dong-ya YUAN, Zhong-dao WU, Zhi-yue LV

RESUMEN

La esquistosomiasis es una importante enfermedad zoonótica producida por parásitos que ocasiona daño severo a seres humanos y animales. El monitoreo y el diagnóstico juegan un rol fundamental en el control de la esquistosomiasis. Sin embargo, las técnicas actuales de monitoreo y diagnóstico de la enfermedad tienen limitaciones. Debido a que aumenta la información del genoma de los parásitos, las técnicas nuevas para la

detección que incorporan secuencias de nucleótidos están recibiendo mayor atención. Estos métodos de detección rápidos, sensitivos y específicos son particularmente importantes en el diagnóstico de infecciones tempranas y de bajo grado y es posible que tengan gran importancia clínica. Este artículo analiza el progreso de la detección de ácido nucleico en el diagnóstico y prevención de la esquistosomiasis, incluyendo aspectos tales como la selección de genes objetivo y el desarrollo y aplicación de métodos de detección de ácido nucleico.

Translated from English version into Spanish by Maria Alejandra Aguada, through
